# Functional health and symptoms in Spain before and during the COVID-19 pandemic

**DOI:** 10.1186/s12889-021-10899-2

**Published:** 2021-05-01

**Authors:** Jens Lehmann, Bernhard Holzner, Johannes M. Giesinger, Andrew Bottomley, Shaad Ansari, Ludwig von Butler, Georg Kemmler

**Affiliations:** 1 University Hospital of Psychiatry I, Medical University of Innsbruck, Anichstraße 35, 6020 Innsbruck, Austria; 2 University Hospital of Psychiatry II, Medical University of Innsbruck, Anichstraße 35, 6020 Innsbruck, Austria; 3 EORTC Department of Quality of Life, Headquarter, Brussels, Belgium; 4 SurveyEngine GmbH, Berlin, Germany

**Keywords:** Health-related quality of life, Quality of life, COVID-19, SARS-CoV-2, Mental health, Spain

## Abstract

**Background:**

The impact of the coronavirus disease (COVID-19) pandemic on wellbeing and health has so far been studied using mostly cross-sectional designs. To place recent findings into context, we compared symptoms and functional health status in two independent samples assessed before and during the COVID-19 pandemic.

**Methods:**

Participants were recruited via an online panel using quota sampling. We assessed symptoms, functional health, and global quality of life with the EORTC QLQ-C30 in two general population samples in Spain (collected in July 2019 and April 2020). We also assessed several COVID-19 related variables, such as adherence to social distancing.

**Results:**

Data from *N* = 1010 participants before the pandemic (mean age 47.1 years, 50.5% female) were compared with data from *N* = 504 participants during the pandemic (mean age 47.1 years, 50.8% female). Participants during the pandemic (vs. before the pandemic) reported lower role functioning and emotional functioning, as well as less symptom burden. A lower degree of social distancing was associated with better functional health and lower symptom burden.

**Conclusion:**

Our findings indicate an impact of the COVID-19 pandemic on functional health and symptom burden in the Spanish general population. The comparison of before and during the pandemic can be used to benchmark results raised only during the pandemic.

**Supplementary Information:**

The online version contains supplementary material available at 10.1186/s12889-021-10899-2.

## Background

The coronavirus disease (COVID-19) is a respiratory infectious disease that is considered a global health threat [1]. The disease is mainly transmitted via the respiratory tract, through direct contact, droplets, or respiratory secretions [2, 3], and the virus can be transmitted even in an asymptomatic stage [4]. Therefore, limiting human-to-human exposure is crucial to combating the spread of this disease [5, 6]. Countries have established public health strategies with varying degrees of population restrictions based on measured incidence rates and other factors such as health system resilience. It is not only crucial to assess the impact of these measures on the disease incidence rates but also important to investigate the psychological and social impact of the disease and restrictions implemented to contain it on a population level [7–9]. The perceived impact of the disease and governmental restrictions are potentially amplified by the constant news coverage of the pandemic and a plummeting economy, which may contribute to an increase in global distress and reduced wellbeing [10–12].

This led to multiple early warnings of a possibly detrimental mental health impact of the pandemic (e.g., [9, 11]). Early results from systematic reviews of mostly cross-sectional studies reported high point-estimate prevalence of anxiety and depressive symptoms in different international populations [13–15]. For example, a first cross-sectional survey of 3480 people in Spain revealed that depressive (18.7%), anxiety (21.6%), and post-traumatic stress disorder (15.8%) symptoms were present in the population [16]. However, as argued by Meda and Slongo [17], caution should be applied when attributing mental health consequences to the COVID-19 pandemic based on cross-sectional data. Instead, a more recently published meta-analysis of longitudinal studies on the psychological impact of COVID-19 lockdowns [18] revealed that the impact is “small in magnitude and highly heterogeneous” and that “lockdowns do not have uniformly detrimental effects on mental health”.

Among the European countries, Spain had particularly high prevalence rates and a high death per capita rate [19]. Despite having one of the best performing health care systems in the world [20], Spain has been placed among the worst affected counties [21]; in part due to a potentially delayed governmental response [21], a shortage of medical equipment and personnel [22], and a vulnerably elderly population [23]. In the first week of March, the number of new infections rose beyond containment and on March 15, a nation-wide state of emergency was declared. Spain consists of 17 autonomous communities, but the response to the pandemic has, at least during the initial phase, been a centralized one; the lockdown imposed during the state of emergency on March 28 affected all residents equally. The government issued a halt to all non-essential activities, businesses, and schools and encouraged remote work wherever possible. For example, Spaniards were not allowed to leave their homes or even go for a walk, unless for essential activities, medical supply, or grocery shopping [24]. Unemployment rose by 9% in March and by 21% in April, as non-essential work and tourism were brought to a standstill [25]. Moreover, the crisis had an effect on the provision of healthcare outside of COVID-19. As many resources were needed for the treatment of patients with COVID-19 and patients may have been hesitant to attend their general practitioners or hospitals, the access to medical care, including even oncology referrals or treatments, was reduced [26].

In this study, we aimed to investigate the impact of these unprecedented circumstances on the Spanish population’s symptoms, functional health, and quality of life (QoL). Similar to some of the studies included in the aforementioned meta-analysis of Prati and Mancini [18], we were able to utilize previously collected Spanish general population data from another project for which assessments were conducted shortly before the COVID-19 outbreak and compared it with a new dataset assessed shortly after the peak of the pandemic in Spain.

Our primary objective was to compare the general population’s levels of symptoms and functional health from before the pandemic with data collected shortly after the peak of the pandemic in Spain. A secondary objective was to analyze the association of self-reported adherence to governmental restrictions, social distancing, job status, and living situation with participants’ health status.

## Methods

**Fig. 1:**
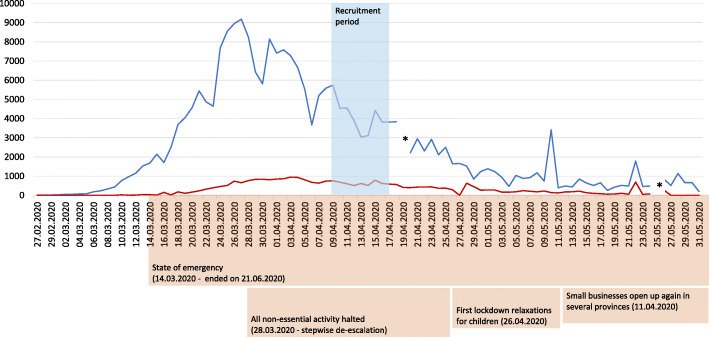
Timeline of the COVID-19 outbreak and assessment period. Shows the number of newly reported infections in blue and the number of newly reported deaths in red; the recruitment period is shaded in blue; data by the European Centre for Disease Prevention and Control [27]; * negative values are not shown but can occur when a country sends a correction to the ECDC, because it had previously overestimated the number of cases/deaths

We compared participants’ health status from two time points: the first one preceding the COVID-19 outbreak in Spain and the second one during the state of emergency declared owing to the pandemic (assessment between April 4 and April 17, 2020). Figure 1 visualizes the timeline of the second assessment mapped alongside the number of new infections and deaths per day in Spain.

### First assessment

The pre-COVID-19 data were available from another research project on health utility evaluations for EORTC QLU-C10D [28, 29] of the Spanish general population. Recruitment took place between July 4 and July 29, 2019, and the survey was completed by *N* = 1010 participants. In addition to the socio-demographic information (developed for this study, see Supplement 1), the survey comprised participants’ evaluations of functional health statuses (health utilities) and a questionnaire on participants’ symptoms and functional health status, the EORTC QLQ-C30 [30]. Only the latter was analyzed in this study.

### Second assessment

Shortly after the peak of the COVID-19 infection rate in Spain, we performed a second assessment, which was constructed analogously to the first. Building on the survey, we asked participants seven additional questions, which were added at the end of the questionnaires. Those questions concerned their current job status (assessing those working remotely and loss of job due to the pandemic), living situation (available outdoor space and size of household in m^2^), concern about the pandemic, if they had been tested for COVID-19, and adherence to social distancing restrictions by the government. Adherence to social distancing was assessed with four possible answer options: (1) “no social distancing,” (2) “moderate social distancing (reduced contact with others, work in the workplace if appropriate),” (3) “heavy social distancing (leaving the house for essential tasks, such as shopping, doctor’s visits, teleworking, if applicable),” (4) “quarantined.” To increase the statistical power for comparisons between low to moderate and high levels of social distancing, options (1) and (2) and options (3) and (4) were combined for the statistical analysis.

#### EORTC QLQ-C30

The EORTC QLQ-C30 [30] is a 30-item cancer-specific questionnaire that assesses symptoms, functional health, and global QoL. It is one of the most widely used questionnaires in clinical trials and practice but can also be used to compare cancer patients’ functional health and symptoms to that of the general population [31–36]. The EORTC QLQ-C30 consists of five functional scales (physical, emotional, social, role, and cognitive), nine symptom scales (fatigue, nausea/vomiting, pain, dyspnea, sleep disturbances, appetite loss, constipation, diarrhea, and financial impact), and a global QoL scale. Scales range from 0 to 100, with high scores on the functional scales indicating better functioning and high scores on the symptom scales indicating higher symptom burden. We decided to exclude the financial difficulties and social functioning scales from the analysis as they are worded specifically for patients (e.g., “Has your physical condition or medical treatment caused you financial difficulties?”). Participants from the general population could be confused about the wording and unsure of how to answer the questions if they do not have a physical condition.

#### Thresholds for clinical importance

Thresholds for clinical importance (TCIs) were recently developed for the QLQ-C30 [37]. These thresholds allow the identification of clinically important problems or impairments in the QLQ-C30 domains. Patient scores below the TCI for functioning scales, or above the TCI for symptom scales indicate clinically important problems or symptoms. For instance, exceeding the TCI for physical functioning might indicate that the person experiences impairments in physical functioning (e.g., walking) that limit him or her in their daily life, that he or she needs help with, or that have caused his or her family/partner to worry. These TCIs were developed using a sample of patients with cancer and have not been validated in the general population so far. However, as general population-specific TCIs are not available in the literature, we relied on these thresholds to indicate what percentage of participants experience significant impairments.

#### Sampling

Sampling was conducted by an online panel research company (https://surveyengine.com/). Respondents (eligible if aged 18 to 80 years and residing in Spain) were members of an online panel who had agreed to participate in such studies. Hyperlinks to the survey were sent to respondents, and after providing informed consent, participants could complete the survey at their leisure. Quota sampling based on national census data [38] was used to best represent the general population regarding age and sex. See Supplement 2 for the detailed age and sex quotas and comparison to the general population. If participants completed the survey, they were compensated with a small payment (~ 0.5€).

### Statistical analysis

All statistical analyses were performed using SPSS, version 24. Prior to the analysis, all metric variables were checked for deviations from normality by investigating their skewness and kurtosis. As the majority of the QLQ-C30 subscales showed skewness values <− 1 or > 1, indicating considerable deviations from normality [39], we employed non-parametric testing for all QLQ-C30 scales. The Mann–Whitney U test was applied for comparing the two samples, with respect to the QLQ-C30 subscales. Chi-square tests were used for group comparisons regarding the prevalence of impairments in the individual domains, as defined by the aforementioned TCIs.

In the second sample (during COVID-19), potential effects of social distancing (no or moderate social distancing vs. heavy social distancing or quarantine) on the QLQ-C30 subscales were investigated by means of the Mann–Whitney U test. The relationships between the restrictions of participants’ work-life and their QOL-C30 scores were analyzed using Kruskal–Wallis tests. Effects of the living situation (two levels) were analyzed by the Mann–Whitney U test. Associations of household size (m^2^ per person) with QLQ-C30 scores were analyzed by Spearman rank correlation coefficients. As the household size significantly correlated with age, we performed an additional analysis with adjustment for age (ordinal regressions with size of household and age as independent variables, and QLQ-C30 scores as dependent variables).

Potential regional effects were tested once by the Kruskal–Wallis test, considering all 17 Spanish regions as distinct factor levels, and once, by correlation analysis, calculating Spearman rank correlation coefficients between the mean number of COVID-19 cases per million inhabitants and QLQ-C30 scores.

Sample size considerations for the second, prospective sample focused on the overall comparison of QLQ-C30 scales between the first and second samples and on subgroup comparisons for the second sample. A sample size of *N* = 1000 in the first sample and of *N* = 500 in the second sample provides 95% power to detect a difference of Cohen’s d = 0.20 in a non-parametric comparison with a Mann–Whitney test (alpha = 0.05, two-sided). In subgroup analyses comparing two groups based on participant characteristics such as job status or social distancing, a total sample size of *N* = 500 provides at least 80% power to detect a difference of d = 0.30, if the smaller group comprised at least 120 participants (alpha = 0.05, two-sided, non-parametric Mann–Whitney test).

## Results

### Sample characteristics

#### Sample 1

For the first assessment, 1625 participants answered the survey. Participants were excluded if they refused to take part (*n* = 17, 1.0%), dropped out before completing the survey (*n* = 236, 14.5%), or were excluded, as their respective quotas were already achieved (*n* = 362, 22.3%). The final sample (*N* = 1010) showed equally distributed sex (49.5% males, 50.5% females) and an age distribution representative of the Spanish general population.

#### Sample 2

Between April 4, 2020, and April 17, 2020, 1129 participants initiated the survey. Participants were excluded if they refused to take part (*n* = 7, 0.6%), dropped out before completing the survey (*n* = 303, 26.8%), or were excluded, as their respective quotas were already achieved (*n* = 315, 27.9%). The final sample (*N* = 504) showed equally distributed sex (49.2% males, 50.8% females) and an age distribution representative of the Spanish general population. Of the participants, *n* = 21 (4.2%) had been tested for SARS-CoV-2, of whom *n* = 14 (2.8%) tested negative, *n* = 2 (0.4%) were awaiting their results, and *n* = 5 (1.0%) tested positive. Table 1 displays the socio-demographic information for both samples.

**Table 1:** Socio-demographic and clinical variables^a^

Variable		Before COVID-19 pandemic (*N* = 1010)	During COVID-19 pandemic (*N* = 504)	Statistics	*p*-value
Age	Mean ± SD	47.10 ± 15.50	47.06 ± 15.46	Z = 0.004	0.997
Sex	Female	510 (50.5%)	256 (50.8%)	χ^2^ = 0.012	0.913
	Male	500 (49.5%)	248 (49.2%)		
Education	No A-levels	230 (22.8%)	123 (24.4%)	χ^2^ = 0.501	0.497
	A-levels or higher	780 (77.2%)	381 (75.6%)		
Marital status	Single	282 (27.9%)	133 (26.4%)	χ^2^ = 2.194	0.533
	Married/partnership	608 (60.2%)	316 (62.7%)		
	Divorced/separated	79 (7.8%)	41 (8.1%)		
	Widowed	41 (4.1%)	14 (2.8%)		
Any chronic diseases		347 (34.4%)	167 (33.1%)	χ^2^ = 0.224	0.636
Work status^b^	Normal work		251 (49.8%)		
	Working remotely		55 (10.9%)		
	Unemployed		57 (11.3%)		
	Lost work due to COVID-19		19 (3.8%)		
	Houseworker		19 (3.8%)		
	Retired		77 (15.3%)		
	In training or student		15 (3.0%)		
	Other		11 (12.2%)		
Living space (in m^2^)^b^	Mean ± SD		111.7 ± 15.46		
No. of people living in household^b^	Mean ± SD		2.98 ± 1.24		
Outside living space available^b^			398 (79.0%)		
Social distancing^b^	Not at all		70 (13.9%)		
	Moderate		60 (11.9%)		
	Strong		217 (43.1%)		
	Quarantined		157 (31.2%)		
Tested for SARS-CoV-2^b^	Not tested		483 (95.8%)		
	Negative result		14 (2.8%)		
	Positive result		5 (1.0%)		
	Results pending		2 (0.4%)		

^a^ Table entries are n (%), if not denoted otherwise; ^b^ variable assessed only in the assessment during COVID-19; *SD* standard deviation

### Effects on QLQ-C30

Table 2 shows the average health status as measured with the QLQ-C30 before and during the COVID-19 pandemic. On the functioning scales, participants during the COVID-19 pandemic reported significantly deteriorated role functioning (i.e., impairments in family or social life) and emotional functioning compared with participants surveyed before the pandemic. Conversely, on the symptom scales, participants during the COVID-19 pandemic reported significantly less fatigue, pain, dyspnea, and appetite loss compared with participants surveyed before the pandemic.

**Table 2:** Health-related quality of life before and during the COVID-19 pandemic

QLQ-C30 subscales	Before COVID-19 pandemic (*N* = 1010)		During COVID-19 pandemic (*N* = 504)		Comparison	
	Mean	SD	Mean	SD	d	*p*-value
Physical functioning	91.3	13.3	92.1	12.3	0.06	0.143
Role functioning	90.6	17.6	86.5↓	23.8	−0.21	0.034
Emotional functioning	77.2	22.3	72.7↓	22.5	−0.20	0.000
Cognitive functioning	90.2	17.2	90.9	16.1	0.04	0.591
Global quality of life	70.5	18.8	69.0	18.2	−0.08	0.120
Fatigue	22.8	19.8	18.1↓	19.2	−0.24	0.000
Nausea and vomiting	3.8	11.8	3.8	10.4	0.00	0.413
Pain	20.1	21.1	16.6↓	19.7	−0.17	0.001
Dyspnea	10.3	19.4	8.0↓	17.7	−0.12	0.015
Sleep disturbances	26.6	26.9	27.3	28.5	0.03	0.929
Appetite loss	10.3	20.6	7.9↓	17.8	−0.12	0.036
Constipation	13.4	22.5	14.2	22.0	0.04	0.276
Diarrhea	7.7	17.7	7.4	16.2	−0.01	0.727

↓ significantly lower at second assessment, *SD* standard deviation

### Percentage of participants with clinically important results

Table 3 shows the percentage of participants with clinically important results for the QLQ-C30 scales before and during the COVID-19 pandemic, as determined by the TCIs. Most notably, during the COVID-19 pandemic, significantly more participants reported clinically important levels of emotional functioning compared with participants before the pandemic. A similar effect was found for role functioning, where a significantly higher percentage of participants during the COVID-19 pandemic reported clinically important results compared with participants before the pandemic.

**Table 3:** Proportion of respondents with clinically important problems in QLQ-C30 subscales before and during the COVID-19 pandemic

	Before COVID-19 pandemic (*N* = 1010)	During COVID-19 pandemic (*N* = 504)	Comparison	
			Chi-square	*p*-value
Physical functioning	17.6%	17.9%	0.02	0.911
Role functioning	5.0%	11.1% ↑	19.60	< 0.001
Emotional functioning	33.7%	40.9% ↑	7.58	0.006
Cognitive functioning	15.1%	13.7%	0.57	0.450
Fatigue	16.7%	14.1%	1.76	0.184
Nausea and vomiting	13.0%	14.7%	0.84	0.359
Pain	39.6%	32.9% ↓	6.38	0.012
Dyspnea	25.3%	19.8% ↓	5.67	0.017
Sleep disturbances	16.3%	18.8%	1.49	0.222
Appetite loss	5.7%	4.0%	2.17	0.141
Constipation	6.9%	6.3%	0.18	0.761
Diarrhea	18.6%	9.6%	0.23	0.630

Thresholds for clinical importance as defined by Giesinger et al. (2019); ↑ = significantly higher percentage of participants exceeding the threshold; ↑ = significantly lower percentage of participants exceeding the threshold

For the symptom scales pain and dyspnea, a significantly lower percentage of participants reported clinically important results during the COVID-19 pandemic than before the pandemic.

### Effects of social distancing and other COVID-19-related variables during the second assessment

Participants reported different levels of social distancing during the second assessment (see Table 4). Participants who reported no or moderate social distancing showed better functioning on the scales for emotional functioning and global health/QoL than participants who reported strong social distancing or quarantine. For the symptom scales, participants who reported no or moderate social distancing showed lower symptom burden on the scales pain and sleep disturbances, than participants who reported strong social distancing or quarantine.

**Table 4:** Effects of social distancing on respondents’ HRQOL

	No/moderate social distancing (*N* = 130)		Strong social distancing/quarantine (*N* = 374)		Comparison (Mann-Whitney U-test)	
	Mean	SD	Mean	SD	d	*p*-value
Physical functioning	93.0	11.0	91.9	12.8	−0.09	0.327
Role functioning	87.3	23.8	86.3	23.9	−0.04	0.488
Emotional functioning	79.2	18.3	70.5	23.4	−0.39	< 0.001*
Cognitive functioning	93.3	12.4	90.1	17.1	−0.20	0.125
Global quality of life	72.2	19.8	67.9	17.5	−0.24	0.008*
Fatigue	15.6	17.6	18.9	19.6	0.17	0.090
Nausea and vomiting	4.0	10.7	3.7	10.2	0.03	0.640
Pain	11.9	18.0	18.2	20.0	0.32	< 0.001*
Dyspnea	7.4	15.1	8.2	18.5	0.05	0.863
Sleep disturbances	20.5	25.7	29.7	29.1	0.33	0.001*
Appetite loss	8.5	19.2	7.8	17.3	−0.04	0.805
Constipation	11.8	19.0	15.1	22.9	0.15	0.259
Diarrhea	6.7	13.4	7.7	16.9	0.06	0.979

*SD* standard deviation; d = Cohen’s d (effects size of strong social distancing/quarantine vs. no/moderate social distancing); * significant at *p* < 0.05

Regarding the current job status (working normally vs. working remotely vs. lost job because of the pandemic), we found significant differences in the emotional and cognitive functioning score (Kruskal–Wallis test, *p* < 0.05): The emotional functioning score was reduced both in persons working remotely and in those who lost their job as a result of the pandemic, and the cognitive functioning score was only reduced in persons who lost their job. Moreover, the availability of outdoor space (vs. no outdoor space) in respondents’ households was associated with significantly higher scores in cognitive functioning, emotional functioning, and overall QoL and with reduced reported levels of pain. Size of household in m^2^ showed significant positive correlations with emotional and cognitive functioning (r Spearman between 0.12 and 0.17, *p* < 0.05) and significant negative correlations with fatigue, pain, dyspnea, sleep disturbances, and appetite loss (r between − 0.09 and − 0.13, *p* < 0.05). However, after adjustment for age, none of these correlations remained significant. No significant regional effects were observed, neither in terms of the mean differences in QLQ-C30 scores between regions nor in terms of the correlations between the mean number of COVID-19 cases per million inhabitants and QLQ-C30 scores.

## Discussion

In this study, we compared participant-reported symptoms and functional health status of two samples of the general Spanish population before and during the COVID-19 pandemic. Participants during the pandemic, as compared with participants before the pandemic, reported lower role and emotional functioning, while also showing a lower symptom burden regarding fatigue, pain, dyspnea, and appetite loss. Those participants who reported to perform no or only moderate social distancing exhibited higher functional status, as well as lower symptom burden than participants who performed at least moderate social distancing.

### Differences in participant-reported health status

Several of our findings merit in-depth discussion. During the COVID-19 pandemic, participants reported higher impairments in emotional functioning than before the pandemic. A first cross-sectional survey of 3480 people in Spain revealed that depressive (18.7%), anxiety (21.6%), and post-traumatic stress disorder (15.8%) symptoms were present in the population [16], and in an Italian survey, 38% of participants reported at least mild psychological distress [40]. However, both surveys used snowball sampling and reported an unbalanced sample. Instead, when comparing our findings to the meta-analysis of longitudinal studies on the psychological impact of COVID-19 lockdowns by Prati and Mancini [18], they show remarkable similarity. In their study, Prati and Mancini report a small, yet significant increase in pooled mental health symptoms (effect size Hedges’ g = 0.17, 95% CI 0.06 to 0.24), specifically for depressive and anxiety symptoms. This conforms to our finding of participants reporting lower emotional functioning (e.g., “Did you feel depressed?”; “Did you worry?”) during the pandemic compared to before the pandemic. Notably, the size of the effect we found (a decrease of d = 0.20 in emotional functioning) is remarkably similar to the aforementioned effect size reported by Prati and Mancini [18].

During the pandemic, participants also reported worse role functioning (i.e., more limitations in their work, daily activities, hobbies, and leisure time activities) reflecting the consequences of the lockdown measures and the rising unemployment rate [25]. However, like for emotional functioning, the absolute effect of the decrease in role functioning was rather small (d = 0.21).

Regarding symptoms assessed with the QLQ-C30, an interesting finding was that that lower levels of fatigue, pain, and dyspnea were reported by participants during the pandemic than participants before the pandemic. Similar to our finding, in the study by Sibley, Greaves et al. [41], participants during COVID-19 exhibited less fatigue than those before COVID-19. Experiencing pain and dyspnea is often dependent on physical activity [42, 43], which was reduced by the curfew. A Spanish study estimated that physical activity in the population was reduced by 20% during the first week of confinement [44]. However, this interpretation must be considered carefully. First, absolute differences were considerably small (effect size d < 0.25). Second, over time, less physical activity could lead to a loss of fitness and a subsequent increase in pain and dyspnea once restrictions are lifted.

### Differences between participants performing and not performing social distancing

Participants that performed no social distancing exhibited better functional health as well as lower symptom burden than participants who performed at least moderate social distancing. One particular finding was that participants practicing a high level of social distancing, compared to participants practicing less social distancing, reported more sleep disturbances. In an Italian sample tested during the lockdown in March 2020, participants reported sleeping longer, but, at the same time, reported a lower quality of sleep [45]. The latter was stronger for participants with higher levels of depression, anxiety and stress. This may explain our finding of more sleep disturbances in participants with higher levels of social distancing. Another international cross-sectional survey found that a third of the *N* = 6882 surveyed participants reported increased sleep disturbances during the pandemic. Among the factors that were associated with poorer sleep quality were a stricter level of quarantine and other pandemic-related factors (e.g., loss of employment or financial strain) [46].

Sleep disturbances can also serve as a sentinel for mental health problems [47, 48] and, accordingly, we also found lower emotional functioning in the participants who practiced more social distancing. A systematic review of psychological health and physical activity during the pandemic indicated that a reduced level of physical activity due to the pandemic was associated with controversial psychological outcomes [49]; a finding that may explain that participants in our study who were practicing more social distancing (and due to the confinement being able to practice less physical activity) reported lower emotional functioning. Conversely, our results are somewhat opposed to the results of a survey in China that found no difference in psychological symptoms, anxiety, or depression, depending on quarantine management [50]. However, the study compared participants under lockdown and not under lockdown and therefore, unlike our study, included participants not directly affected by the regulations. It also did not assess the extent of adherence to the quarantine measures imposed, as we did in our study. Rather, the measured dissatisfaction with the control measures predicted negative psychological consequences; a variable we did not assess in our study.

A rapid review by Brooks et al. [7] presents various non-COVID related sources that link quarantine and psychological distress and symptoms of post-traumatic stress disorder. This aligns with the aforementioned findings that those who performed less social distancing showed higher emotional functioning and global health and QoL. However, the assessment of social distancing is likely confounded with several other factors (e.g., rural vs. urban localization, attitude towards the health system). We also assessed participants during the most restrictive phase of the nation-wide lockdown in Spain when the perceived thread by COVID-19 was probably high [10, 16]. Those participants that were most afraid of the disease might also be the ones most adherent to the restrictions and, consequently, the most affected.

### Strengths and limitations

A strength of this study is that we were able to use QLQ-C30 data from a few months before the COVID-19 pandemic. In conjunction with the consistent methodology applied in both assessments (before and during the pandemic), this allowed us to evaluate changes in the population’s symptoms and functional health. However, due to the ad-hoc nature of our study, we were not able to re-assess the same participants from the first assessment, for our second assessment. Consequently, this study was not a longitudinal observation of the same participants but a comparison of two cross-sectional samples of the Spanish population, which needs to be considered when interpreting the results. However, our results are well in line with longitudinal studies on the psychological impact the lockdown that have since been published and the meta-analysis by Prati and Mancini [18] confirms the general direction of our results.

Another caveat of methodological nature is that the QLQ-C30 and its TCIs were constructed to measure symptoms and functional health status in patients with cancer. Even though questions of the QLQ-C30 are aimed to measure problems of patients with cancer, the questionnaire can be completed by members of the general population, as most questions are broadly applicable (e.g., “Do you have any trouble taking a long walk?”). Moreover, the QLQ-C30 has been successfully used in general population samples to establish normative datasets in several countries [31–36]. Most notably, Mols et al. [51] showed that the QLQ-C30 can be used to measure change over time in the general population.

Finally, while the quota sampling we applied provides representability in terms of age and sex, the sample may differ from a true population sample as participants were recruited from an online survey pool. Panel data are prone to selection bias regarding participants socio-economic status (higher participation rates by participants with lower economic income) and access to digital technologies (i.e., only participants with internet access can participate). As we did not measure participants’ socio-economic status, we cannot rule out that our samples are biased in those regards.

## Conclusions

This study compares functional health, symptom burden, and global QoL in two samples of the Spanish general population before and during the COVID-19 pandemic. Due to the consistent mode of data collection and careful sampling, changes in scores most likely represent population-level changes over time. Analysis and comparison of our findings with similar studies, including longitudinal studies, indicate that the Spanish population during the initial weeks of the COVID-19 pandemic exhibited impairments in terms of functional health, while also displaying several minor reductions in symptom burden. All effects were small in magnitude. Our study therefore suggests that during the first weeks of the pandemic, there were no major psychological impacts or impacts in QoL in the Spanish population. Considering the substantial heterogeneity of findings on an international level and considering that effects might change over time, further research is warranted to monitor the long-term impact of the COVID-19 pandemic on a population level.

## Supplementary Information

**Additional file 1: Supplement 1.** ‘Supplement (1) to “Functional health and symptoms in Spain before and during the COVID 19 pandemic” by Lehmann et al. 2021 at BMC Public Health’, This supplement gives an English translation (original language: Spanish) to the sociodemographic questionnaire that was constructed for this study. Questions or answer options that are marked with grey color were present only in the second assessment (in 2020).

**Additional file 2: Supplement 2.** ‘Supplement 2 to “Functional health and symptoms in Spain before and during the COVID-19 pandemic” by Lehmann et al. 2021 at BMC Public Health’, This supplement details the sampling strategy for both samples. The sampling strategy was based on UN data from 2017 at: UNdata | record view | Population by age & sex and urban/rural residence. [2020 Jun 24]. Available from: http://data.un.org/Data.aspx?d=POP&f=tableCode%3a22.

## Data Availability

The authors thank the European Organization for Research and Treatment of Cancer for permission to use the data from the EORTC (EORTC QLG grant number 001/2018) for this research. The contents of this publication and methods used are solely the responsibility of the authors and do not necessarily represent the official views of the EORTC. The datasets analyzed in the study at hand are available upon reasonable request from the EORTC. Please use the Data Sharing form available through the EORTC website (https://www.eortc.org/data-sharing/).

## Abbreviations

EORTC: European Organization for Research and Treatment of Cancer
COVID-19: Coronavirus disease
QoL: Quality of Life
TCI: Threshold for clinical importance
