# Supplementary material for: Functional health and symptoms in Spain before and during the COVID-19 pandemic
Source: BMC Public Health. 2021 May 1;21:837. doi: 10.1186/s12889-021-10899-2 (PMC8087887; doi:10.1186/s12889-021-10899-2)
Supplement: Supplementary file 1 — Additional file 1: Supplement 1. ‘Supplement (1) to “Functional health and symptoms in Spain before and during the COVID 19 pandemic” by Lehmann et al. 2021 at BMC Public Health’, This supplement gives an English translation (original language: Spanish) to the sociodemographic questionnaire that was constructed for this study. Questions or answer options that are marked with grey color were present only in the second assessment (in 2020). [file 12889_2021_10899_MOESM1_ESM.pdf]

Supplement (1) to "Functional health and symptoms in Spain before and during the COVID-19 pandemic" by Lehmann et al. 2021 at BMC Public Health

This supplement gives an English translation (original language: Spanish) to the sociodemographic questionnaire that was constructed for this study. Questions or answer options that are marked with grey color were present only in the second assessment (in 2020).

Question	Possible answers
Are you male or female?	Male<br>Female
How old are you (in years)?	(participants enter an integer number)
What is your highest education or training?	Compulsory or less<br>Spanish baccalaureate<br>Vocational education<br>Non-university diploma<br>University degree<br>Postgraduate degree
Do you have any, or have you had any of the following (tick all that apply)?	Asthma, emphysema or chronic bronchitis<br>Arthritis or rheumatism<br>Cancer diagnosed in the last 3 years<br>Diabetes<br>Digestive problems (such as ulcer, colitis or gallbladder disease)<br>Heart problems (such as angina pectoris, congestive heart failure or coronary artery disease)<br>HIV/AIDS<br>Renal disease<br>Liver problems (such as cirrhosis)<br>Stroke<br>Mental illness (such as depression)<br>Stroke<br>None of the above
What is your relationship status?	Single<br>Married<br>Divorced<br>Widowed<br>Long-term relationship (but not married)
Do you live alone?	Yes<br>No
Do you live with your children?	Yes<br>No
Do you live with your spouse/partner?	Yes<br>No

Do you live with your parents?	Yes<br>No
Do you live with other family?	Yes<br>No
What is your current employment status?	Employed (full time)<br>Employed (full time, currently teleworking)<br>Employed (part time)<br>Employed (part time, currently teleworking)<br>Unemployed<br>Lost my job due to COVID-19<br>Houseworker<br>Retired<br>On sick leave<br>In training or student<br>Other
How many square meters does your apartment/house have (including garden or balcony)?	(participants enter an integer number)
How many persons live in your apartment/house?	(participants enter an integer number)
Does your apartment/house have a garden or balcony?	Yes<br>No
Social distancing	No social distancing<br>Moderate social distancing (reduction of contact with other people, work at the workplace if applicable)<br>Strong social distancing (I only leave home for essential tasks such as shopping, doctor's visits, teleworking if necessary)<br>I am in quarantine
Are you burdened by the current situation caused by Covid-19?	Not at all<br>A little<br>Quite a bit<br>Very much
Have you been tested for Covid-19?	No<br>Yes, with a negative result (I don't have COVID-19)<br>Yes, with a positive result (I have COVID-19)<br>Yes, but I am still waiting for the results
