# Supplementary material for: Functional health and symptoms in Spain before and during the COVID-19 pandemic
Source: BMC Public Health. 2021 May 1;21:837. doi: 10.1186/s12889-021-10899-2 (PMC8087887; doi:10.1186/s12889-021-10899-2)
Supplement: Supplementary file 2 — Additional file 2: Supplement 2. ‘Supplement 2 to “Functional health and symptoms in Spain before and during the COVID-19 pandemic” by Lehmann et al. 2021 at BMC Public Health’, This supplement details the sampling strategy for both samples. The sampling strategy was based on UN data from 2017 at: UNdata | record view | Population by age & sex and urban/rural residence. [2020 Jun 24]. Available from: http://data.un.org/Data.aspx?d=POP&f=tableCode%3a22. [file 12889_2021_10899_MOESM2_ESM.pdf]

Supplement 2 to "**Functional health and symptoms in Spain before and during the COVID-19 pandemic**" by Lehmann et al. 2021 at BMC Public Health

This supplement details the sampling strategy for both samples. The sampling strategy was based on UN data from ***2017*** *at:*
UNdata | record view | Population by age & sex and urban/rural residence. [2020 Jun 24]. Available from: http://data.un.org/Data.aspx?d=POP&f=tableCode%3a22.

Age categorized in the Spanish general population 2017

Age group	male (relative)	female (relative)	Total
**18-29**	8.1%	7.8%	15.0%
**30-39**	9.2%	9.2%	17.1%
**40-49**	11.0%	10.7%	22.0%
**50-59**	9.4%	9.5%	19.6%
**60-69**	6.8%	7.4%	14.8%
**70-80**	5.0%	6.1%	11.5%
**Total**	49.4%	50.6%	100.0%

Age categorized in the first sample (Pre-COVID-19)

Age group	male (absolute)	male (relative)	female (absolute)	female (relative)	Total
**18-29**	82	8.1%	81	8.0%	16.1%
**30-39**	92	9.1%	92	9.1%	18.2%
**40-49**	109	10.8%	107	10.6%	21.4%
**50-59**	96	9.5%	96	9.5%	19.0%
**60-69**	69	6.8%	74	7.3%	14.2%
**70-80**	52	5.1%	60	5.9%	11.1%
**Total**	500	49.5%	510	50.5%	100.0%

Age categorized in the second sample (Peri-COVID-19)

Age group	male (absolute)	male (relative)	female (absolute)	female (relative)	Total
**18-29**	41	8.1%	39	7.7%	15.9%
**30-39**	46	9.1%	48	9.5%	18.7%
**40-49**	55	10.9%	54	10.7%	21.6%
**50-59**	47	9.3%	48	9.5%	18.8%
**60-69**	34	6.7%	37	7.3%	14.1%
**70-80**	25	5.0%	30	6.0%	10.9%
**Total**	248	49.2%	256	50.8%	100.0%
